# Ghanaians Might Be at Risk of Excess Dietary Intake of Potassium Based on Food Supply Data

**DOI:** 10.1155/2018/5989307

**Published:** 2018-10-17

**Authors:** David Oscar Yawson, Michael Osei Adu, Benjamin Ason, Frederick Ato Armah, Emmanuel Boateng, Reggie Quansah

**Affiliations:** ^1^Department of Soil Science, School of Agriculture, College of Agriculture and Natural Sciences, University of Cape Coast, Ghana; ^2^Department of Crop Science, School of Agriculture, College of Agriculture and Natural Sciences, University of Cape Coast, Ghana; ^3^Soil Research Institute, Council for Scientific and Industrial Research, Accra, Ghana; ^4^Department of Environmental Science, School of Biological Science, College of Agriculture and Natural Sciences, University of Cape Coast, Ghana; ^5^Department of Biological, Environmental & Occupational Health Sciences, School of Public Health, College of Health Sciences, University of Ghana, Accra, Ghana

## Abstract

The World Health Organization (WHO) has highlighted the beneficial role of adequate intake of potassium (K) in combating the global burden of noncommunicable diseases (NCDs), mainly hypertension and cardiovascular diseases. Diets are the main source of K supply to humans and can contribute to both K deficiency (hypokalemia) and excess (hyperkalemia). While global attention is currently devoted to K deficiency, K excess can be even more dangerous and deserves equal attention. The objectives of this paper were to (i) estimate the K intake of Ghanaian population using food supply and food composition data and (ii) compare this estimate with the WHO-recommended requirement for K in order to assess if there is a risk of inadequate or excess K intake. Food supply data (1961–2011) were obtained from the Food Balance Sheet (FBS) of the Food and Agriculture Organization of the United Nations to derive trends in food and K supply. The average food supply in the FBS for 2010 and 2011 was used in assessing the risk of inadequate or excess dietary intake of K. The K content of the food items was obtained from food composition databases. Based on 2010-2011 average data, the K supply per capita per day was approximately 9,086 mg, about 2.6-fold larger than the WHO-recommended level (3,510 mg). The assessment suggests a potentially large risk of excess dietary K supply at both individual and population levels. The results suggest the need for assessing options for managing K excess as part of food security and public health strategies. The results further underscore a need for assessment of the K status of staple food crops and mixed diets, as well as K management in food crop production systems in Ghana.

## 1. Introduction

Adequate mineral nutrition is a major component of food security strategies. Potassium (K) is an essential element which plays crucial roles in the nutrition and health of plants, animals, and humans. Potassium is known to activate over 60 enzymes in plants, promotes photosynthesis, and plays a role in stomata opening, use of nitrogen, transport of assimilates, and microbial population in the rhizosphere [1–3]. Major roles of K in humans and animals include maintenance of water balance, osmotic pressure, and acid-base balance, activation of enzymes, and mediation of carbohydrate and protein metabolism. More importantly, potassium plays a crucial role in the regulation of neuromuscular activity and heartbeat [4, 5].

Globally, the adverse health outcomes of inadequate intake of vitamins and mineral elements (known as the “hidden hunger”) have received tremendous attention [6]. Similarly, the global burden of noncommunicable diseases (NCDs) has directed attention to the role of K in these diseases [7, 8]. There is a strong evidence of association between low K intake and increased risk of a number of NCDs, including hypertension, cardiovascular disease, chronic kidney stone formation, and low bone-mineral density [5, 9–13]. Low dietary intake of K can result in low serum K concentration, a situation referred to as hypokalemia. Conversely, high serum K concentration (>5.5 mmol/L) or hyperkalemia [14] can result from high dietary supply, problems with K excretion, and imbalance between intracellular and extracellular concentrations [15]. Compared to hypokalemia, hyperkalemia is quite rare but generally more serious and less well tolerated [15–17]. Hyperkalemia can result in a feeling of tiredness or weakness, numbness or tingling, breathing difficulties, chest pains, and palpitations or irregular heartbeats. In extreme cases, paralysis or heart failure can occur [14, 15]. Because the reported average K intake from diets in several countries is below the recommended threshold, the need for increased dietary intake of K-rich diets has been highlighted and efforts are being promoted globally [12, 18].

While the instrumental role of adequate K intake, through food, and its cost-effectiveness in combating the global burden of NCDs are attracting priority attention [12], the risk of excess K intake from diets and its associated adverse health outcomes are not being given comparable priority considerations because this condition is thought to be rare compared to K deficiency [15]. Diets are the main source of K supply in humans. The K content of food components largely derives from the soils on which feed and food crops are grown and the capacity of crops for K uptake. Yet, the K status of soils, K uptake, and fertilizer management in most agroecosystems continue to receive less attention, and this is particularly so in Ghana [3, 4]. The objective of this paper was, therefore, to estimate the dietary supply of K and the risk of inadequate or excess dietary supply of K in adult Ghanaian population using food supply and composition data.

## 2. Methods

### 2.1. K Supply from Foods

Prevalence of K deficiency can be assessed directly via the analysis of urine or blood samples. In the absence of such analysis and for larger population size, the deficiency of K can be quantified via food surveys or dietary analysis using food composition data [19] even though food surveys data can be biased by systematic misreporting and behavioural change [20]. Where there is paucity of data on representative food surveys or food composition tables, as is the case for Ghana, alternative sources of data such as the Food Balance Sheet (FBS) provided by the Food and Agriculture Organization (FAO) can be used to indirectly quantify the adequacy of K intake as has been done in similar studies (e.g., [21, 22]). Hence, the current study used the FBS data to indirectly quantify the risk of inadequate K intake in Ghana. A FBS provides a snapshot of the supply and uses of about 92 food items/groups for each of the FAO member countries during a given reference period [23]. The FBS has supply and utilization sides. For a given reference period and food item, total supply is the sum of total domestic production and imports, adjusted to changes in stocks that might have occurred since the beginning of the reference period. On the utilization side, the total supply of the given food item is decomposed into quantities exported, used for animal feed and seed, processed for food and nonfood uses, losses, and the fraction available for human consumption [23, 24]. The fraction of supply of the food item available for human consumption is divided by the total population of a given country to obtain the *per capita supply*. Thus, the FBS does not directly provide information on food consumption but on food availability, which was used as a proxy for consumption in the current study.

The average food supply per person for the latest years (2010 and 2011) in the FBS was computed. This was done to capture the minimum interannual variation in food availability or consumption. Food items were selected from the FBS based on the kg food supply per person. The dietary K supply per person was estimated as the product of per capita food supply (based on the FBS) and the K content of the food items [6, 25]. The K content or supply of each food component was calculated using the corresponding conversion factors for the edible fraction provided in the food composition table. The K contents of the food components (except for cocoa and products, oats, crustaceans, cephalopods, and other molluscs) were obtained from the West African Food Composition Table [26]. The K contents of the food items that were not found in the West African Food Composition Table [26], such as cocoa and products, were obtained from the United States Department of Agriculture-Agricultural Research Service (USDA-ARS) Nutrient Database for Standard Reference [27]. This method has been applied previously in studies that estimated the adequacy or otherwise of minerals in the diets of populations in some countries [6, 21, 25, 28].

To build the final database of K contents of selected food items, food items were excluded if the product of supply and K content was zero or if that particular food component is not known to be widely or commonly consumed in Ghana according to local knowledge. In the food composition databases, effort was made to identify the categories of food items that best matched those in the FBS [6]. Where two or more categories of the same food items are consumed in Ghana according to local knowledge, an average K content was computed to represent that food item. The total K supply (or intake) per person was calculated as the sum of the products of food supply and K composition of all the food items as described earlier. All K contents or concentration data are expressed as mg 100 g^−1^ fresh weight edible portion. To be consistent with the FBS units, the K contents were multiplied by 10 to obtain K supply in mg·kg^−1^ food intake. The per capita food supply and associated K supply for the period 1961–2011 were computed using the FBS and the food composition table, with a similar approach as described earlier, to obtain the trends.

### 2.2. Adequacy of K Supply from Food

The likely risk of inadequate dietary supply of K was assessed at the individual level, and then the prevalence of deficiency at the population level was estimated using the EAR cut-point approach [6, 25, 29]. A detailed description of this approach and its strengths and assumptions are provided in the Food and Nutrition Board [29]. Due to paucity of information, the recommended K intake for adults of 3510 mg·K per person per day [12] was used in the current study as the reference nutrient intake (RNI). The RNI represents the intake level of a mineral which meets the nutrient requirements of 97.5% apparently healthy individuals in a population group for a given age and sex [6]. Again, due to paucity of information, we used a standard conversion of RNI 1.2∗EAR (as [6] used for Mg and explained by [30] to convert the RNI to an estimated average requirement (EAR) of 2925 mg).

To assess the risk of inadequacy at the individual level, the EAR value was used to represent the “required mean K intake” (*r*), while the total K supply (based on the FBS and food composition data) represented the “observed mean intake” (*y*). The difference between *y* and *r*, *D*, gives an initial impression of the adequacy or otherwise of K intake per person. To allow a probability of correct conclusion on the adequacy of intake, the magnitude and direction (positive or negative) of the ratio of *D* and its standard deviation (SD_*D*_) was estimated [29]. The SD_*D*_ represents the daily variation in individual intake of K. To calculate the SD_*D*_, the standard deviation of the required intake (SD_r_) was estimated at 10% and 15% [29], while the pooled standard deviation of the observed intake (SD_i_) for adult males and females was obtained from reference tables [29] due to lack of national-level data. The SD_*D*_ was then calculated using the procedure in [29]:

(1) $$\begin{array}{c} SD_{D}={\left[SD_{\text{i}}^{2}/n+SD_{\text{r}}^{2}\right]}^{1/2}, \end{array}$$

where *n* is the number of days of observed intake data.

Subsequently, the ratio of *D* to SD_*D*_ was computed for each case (at 10 and 15% for adult males and females) to obtain the probability of correct conclusion regarding the adequacy or otherwise of individual intake (based on the interpretation table in [29]).

The EAR cut-point method was used to estimate the likely prevalence of inadequate intake at the population level. In the EAR cut-point method, a normal distribution of daily intake among the population was expected. The proportion of the population at risk of inadequate intake is assumed to be equivalent to the proportion with intake below the EAR [29]. Because we only had a point estimate of dietary K supply and following an approach used in some previous studies (e.g., [6, 25]), daily K intake in the population was assumed to have a normal distribution, centred on the mean dietary supply and with a coefficient of variation (CV) of 25% or 30%. Based on this, the prevalence of inadequate K intake was estimated using the average of 2010 and 2011 population provided in the FBS which was used to calculate the per capita food supply.

## 3. Results

### 3.1. Contribution of Food Components

The current study included 46 food items in the Food Balance Sheet (FBS) for Ghana (Table 1). The food item coffee and products (mainly instant powder coffee) had the largest K content (3640 mg), followed by mixed ground spices with the K content of 1040 mg. “Meat, other” (mainly game meat) had the third largest K content (923 mg), while “sugar (raw equivalent)” had the least (2 mg). The top three food items with the largest K content (i.e., instant powder coffee, mixed ground spices, and game meat) were consumed in very low quantities in Ghana between 2010 and 2011 according to the FBS. Hence, these contributed less to the overall dietary K supply.

**Figure 1:**
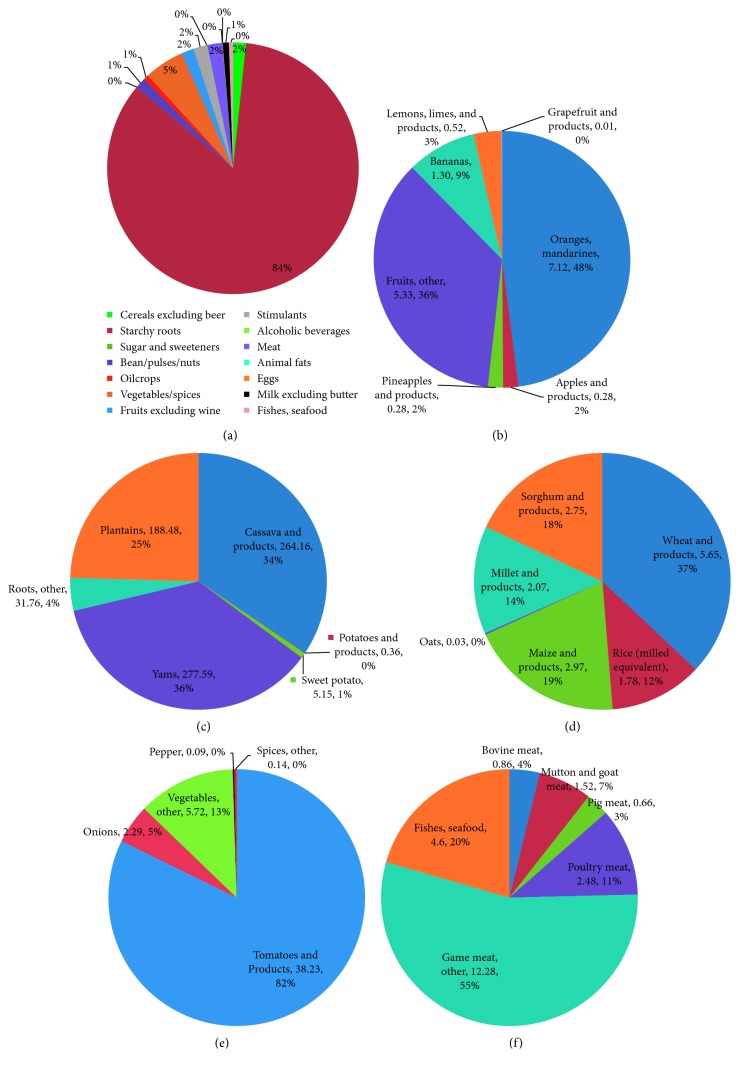
Percentage contribution of main food items to total K supply for Ghana based on 2010-2011 average food supply data: (a) main food items; (b) subcomponents of the food item “fruits”; (c) subcomponents of the food item “starchy roots”; (d) subcomponents of the food item “cereals”; (e) subcomponents of the food item “vegetables”; (f) subcomponents of the food item “meat, fishes, and seafood.” All absolute values of daily per capita K shown are in ×10 mg.

Total K intake from food supply per person was estimated at 9,086 mg per day (Table 1). The top five food items consumed in large quantities were (in order of importance) cassava and products, yams, plantains, roots (other), and rice (milled equivalent). The K supply from these food items was 2641.6, 2775.9, 1884.8, 317.6, and 17.8 mg per capita per day, respectively (Table 1). These top five food items contributed approximately 89% of total dietary K supply. Of the total dietary K supply, starchy roots contributed 84%, while vegetables contributed only 5% (Figure 1(a)). The rest contributed approximately 2% or less. Oranges and mandarines contributed 50% of the total contribution of fruits (Figure 1(b)). Of the starchy roots, yams and cassava contributed the largest (Figure 1(c)). Wheat and products contributed the largest among the cereals (Figure 1(d)), while tomato and products contributed 82% of the total contribution from vegetables (Figure 1(e)). “Game meat, other” contributed 55% of the total contribution of meat, fishes, and seafood.

### 3.2. Trends in K Intake

**Figure 2:**
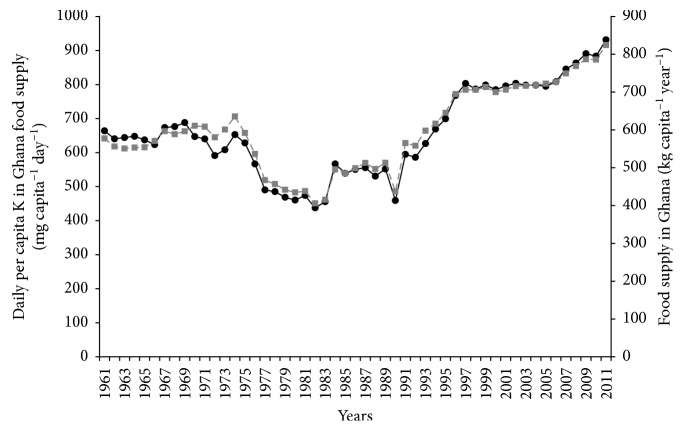
Trend in daily per capita K in food supply and per capita food supply in Ghana for the period 1961–2011. Mean daily capita^−1^ K content (primary axis, filled black circles with continuous line); food supply in Ghana (secondary axis, filled grey squares with broken line).

Between 1974 and 1983, dietary supply of K declined sharply from around 6000 mg to around 4500 mg per capita per day (Figure 2). Thereafter, K intake from food supply increased substantially and reached a plateau around 1989. However, from 1991, K intake from food supply increased sharply and consistently with food supply up to 2011.

### 3.3. Risk of Excess K Intake

The average dietary K supply per person for 2010 and 2011 was estimated at 9,086 mg per day. The estimated variations in individual daily intake (SD_*D*_ at both 10% and 15%) were large for the different sex and age categories considered (Table 2). Similarly, the *D*/SD_*D*_ ratios (at both 10% and 15%) were large and positive. According to the interpretation tables provided by the Food and Nutrition Board [29], these large, positive ratios suggest a 98% probability that the usual dietary K intake of the individual is far in excess of the recommended level, indicating risk of excess. This potentially large risk of K excess at the individual level suggests a potentially large probability of excess supply at the population level. Using the EAR cut-point method with a CV of 25% and 30% (based on a population of 24,542 million) resulted in a risk of deficiency for only 104,000 and 348,000 people, respectively.

## 4. Discussion

Potassium (K) is largely supplied to humans from diets and is highly absorbable (about 85–90%). Based on food supply and composition data, average dietary K intake at both individual and population levels in Ghana was about 2.6-fold larger than the level recommended by WHO [12]. This suggests a potentially large risk of excess dietary supply of K amongst adult Ghanaian population for the years under consideration. The large, positive *D*/SD_*D*_ ratios suggest a 98% probability that the usual dietary K intake of the individual is far in excess of the recommended level [29]. The EAR cut-point method also suggested that only a few people might have inadequate dietary K supply. The results in the current study rectify those reported earlier in Yawson et al. [31]. On the contrary, the K intake in several countries has been found to be below recommended levels [12]. This realization, together with the potential role of K deficiency in NCDs, has directed attention to the urgent need to assess and manage dietary supplies of K in human populations [12]. The current study shows that the risk of excess K intake and its associated health outcomes, although rare, need to be given similar attention, especially in jurisdictions where starchy roots and tubers constitute the bulk of diets. Hyperkalemia, just like hypokalemia, affects the cardiac, neuromuscular, and gastrointestinal organs and is less tolerated than hypokalemia [15]. In extreme situations, hyperkalemia can result in sudden death from impaired cardiac conduction [14, 15].

In humans, the bulk of K (about 98%) is stored in intracellular spaces, largely in muscles [17]. Maintenance of a normal intracellular-extracellular ratio is crucial for the healthy functional roles of K. Imbalance in intracellular and extracellular K concentrations results from high K supply, transcellular shifting, and poor K excretion [15]. While severe symptoms of high serum K concentration might occur only at or above 7 mmol·L^−1^, the rapid rate of rise in extracellular K concentration is more dangerous than the slow rate of rise [32]. It has been estimated that while a loss of 200–400 mEq K from the body would reduce serum K concentration by about 1 mEq·L^−1^, 100–200 mEq excess supply would increase serum concentration by about 1 mEq·L^−1^ [17]. This disproportionate increase in the serum K concentration indicates that high dietary supply of K can have rapid and potentially fatal or health-threatening hyperkalemia [15], especially in those with underlying health conditions. Excretion is a major pathway for controlling high serum K concentration [5]. Thus, those with impaired K excretion and high dietary K intake can rapidly suffer the adverse consequences of hyperkalemia.

The dietary source of potassium largely depends on the type of food consumed in large quantities and widely by the population and the K status and fertilizer management of the soils on which crop plants for human and animal feed are grown. The results in the current study show that yams, cassava, and plantains constituted the bulk of diets and K supply. This suggests that, in jurisdictions where starchy roots and tubers constitute the bulk of diets, the population could be at risk of excess K supply. This, in turn, directs attention to K management in food crop production, as well as the quality of diets. Fufu and gari are the cassava-based diets commonly consumed in Ghana, while yams and plantains are commonly consumed in their cooked form without further processing (locally known as “ampesi”). While cassava is more widely consumed in larger quantities than yams in Ghana and is a good source of K, cassava is largely grown on marginal lands by smallholder farmers, with almost zero fertilizer input. However, cassava might benefit from K supply from NPK fertilizers applied to other crops in mixed cropping systems. While external K input has a lower priority in Ghana, especially in sole roots and tuber production systems, roots and tubers are heavy K feeders and can rapidly reduce the K supply of even K-rich soils after a few years of continuous cultivation [3]. Furthermore, even though the food composition table used in the current study was produced in the 70s, cassava and yam seem to have the ability to mobilize and concentrate K even when grown on marginal soils. There is the need for national assessment of the current K status of these roots and tubers and the soils on which they are grown, as well as K in mixed diets.

With quality of diets, the deliberate consumption of fruits and vegetables is only beginning to increase due to health awareness programmes, but even this is constrained by cost, availability, and traditional eating habits. The consistent patterns of food and K supplies (Figure 2) suggest stability in the consumption of the main K-supplying foods in large quantities over time. The gradual increase in the consumption of fruits and vegetables (which have lower K content compared to roots and tubers), together with the rising consumption of processed foods and westernized diets, especially in urban centres, might help lower dietary supply of K even though processed food could increase sodium intake. Moreover, in urban settings, rice (which is lower in K than starchy roots) is increasingly becoming the dominant staple [33], a situation that might result in large contribution of rice to dietary energy but low contribution to K intake in Ghana. There is the need for empirical studies on K contents of Ghanaian food crops (especially staples) and mixed diets and their relationship with adverse health outcomes of hyperkalemia, especially in those with renal or poor K excretion conditions.

The limitations of the approach adopted in the current study have been acknowledged by previous studies (e.g., [6, 25]). The accuracy of data in the FBS and the food composition databases will affect the accuracy of the current estimate. The data fed to the FAO FBS might be unreliable due to Ghana's poor data collection and aggregation on food production, import, and export. There can also be the issue of underreporting regarding the scale of consumption of some food items. For example, Ghanaians are known to consume large quantities of game meats (bush meat) and other nontimber forest products (NFTPs) which are rich in K, yet this is likely underestimated in the FBS. Similarly, Ghanaians are known to consume appreciable amounts of “molluscs, other,” mainly snails and squids, but the FBS does not report food items that are not commercially declared. In Ghana, bush meat is the second most widely eaten meat after chicken [34, 35]. Key examples of bush meat and other NFTPs commonly eaten in Ghana include grasscutters, antelopes, rats, bats, snails, mushrooms, and honey [36]. In 2014, the Wildlife Division of the Forestry Commission of Ghana estimated the annual domestic trade in bush meat alone at US $140 million [37]. While this estimate excludes nontraded (commercially undeclared) bush meat, it suggests that consumption of bush meat can be quite high in Ghana. Similarly, coastal communities have access to a range of fishes at different periods that might not be reported or captured in the FBS. Due to the underreporting of these food items not considered to be “mainstreamed” or obtained from other sources such as subsistence farming or from the wild, the estimated risk of excess K intake can be substantially underestimated. Hence, the result here must be interpreted with caution as it might not reflect the true dietary K intake in the population. However, the results point to a potentially large risk of excess K supply due to large consumption of starchy roots and tubers, a situation that warrants further investigation.

## 5. Conclusion

The risk of K deficiency is beginning to get attention due to the role of K in the global burden of noncommunicable diseases such as hypertension and cardiovascular disease. Potentially, K excess (although rare) can be even more dangerous than K deficiency in humans. Results from the current study suggest that the risk of K excess, especially in jurisdictions where starchy roots and tubers constitute the bulk of diets, deserves equal attention. Based on FBS data and food composition databases, the current study shows potentially a large risk of excess dietary K intake at both individual and population levels among adult Ghanaian population. Total dietary K supply was about 2.6-fold larger than that recommended by WHO. Only a small fraction of the population was found to be at risk of K deficiency according to the EAR cut-point method. Cassava and yams contributed the bulk of dietary K supply. While the result in the current study ought to be interpreted with caution due to limitations of data from the FAO Food Balance Sheet and food composition databases, it provides indications for policy and research attention. The findings suggest the need for empirical assessment of the K status of staple food crops (especially starchy roots and tubers) and mixed diets and K management in food crop systems in Ghana. Furthermore, studies are required on the relationships between food consumption and serum K concentration in adult Ghanaian population to validate the results in the current study. The results in the current study also suggest the need for studies on dietary K supply in similar jurisdictions where starchy roots and tubers constitute the bulk of diets, especially where renal problems are becoming increasingly prevalent.

## Figures and Tables

**Table 1:** Estimated 2010-2011 average food and K supply per person of 46 edible food items based on the Food Balance Sheet (FBS) and food composition data for Ghana.

Food category	Commonly eaten food/product in Ghana	K content (mg/100 g)	Food supply (kg/yr)	K supply (mg/yr)	K supply (mg/day)
Wheat and products	Bread, wheat, white	117.00	17.64	2063.30	56.5
Rice (milled equivalent)	Rice, white, polished, boiled^*∗*^ (without salt)	20.10	32.40	651.01	17.8
Maize and products	Maize, white, stiff porridge^*∗*^ (without salt)	40.00	27.11	1084.20	29.7
Oats^*∗*^	Oats, regular and quick, unenriched, cooked with water (includes boiling and microwaving), without salt	70.00	0.16	10.85	0.3
Millet and products	Millet, whole grain, boiled^*∗*^ (without salt)	124.00	6.11	757.02	20.7
Sorghum and products	Sorghum, whole grain, boiled^*∗*^ (without salt)	122.00	8.24	1004.67	27.5
Cassava and products	^a^	418.58	230.35	96417.12	2641.6
Potatoes and products	Snack, potato chips, made from dried potatoes, plain	637.00	0.21	130.59	3.6
Sweet potatoes	Sweet potato, yellow, boiled^*∗*^ (without salt)	369.04	5.10	1880.25	51.5
Yams	Yam tuber, boiled^*∗*^ (without salt)	687.00	147.48	101318.76	2775.9
Roots, other	^b^	313.43	36.99	11593.86	317.6
Sugar (raw equivalent)	Sugar	2.00	11.60	23.19	0.6
Beans/Peas^*∗*^	^c^	300.49	0.72	215.60	5.9
Soyabeans^*∗*^	^d^	573.26	0.02	8.60	0.2
Groundnuts (shelled equivalent)^*∗*^	^e^	723.74	5.97	4317.13	118.3
Coconuts including copra	^f^	379.88	5.86	2224.17	60.9
Tomatoes and products	^g^	616.00	22.66	13955.48	382.3
Onions^*∗*^	^h^	133.95	6.23	834.49	22.9
Vegetables, other^*∗*^	^i^	241.43	8.65	2088.33	57.2
Oranges, mandarines	Orange, raw	121.18	21.46	2599.92	71.2
Lemons, limes, and products^*∗*^	^j^	114.34	1.67	190.95	5.2
Grapefruit and products^*∗*^	^k^	96.18	0.03	2.89	0.1
Bananas^*∗*^	^l^	238.40	1.99	474.42	13
Plantains^*∗*^	^m^	524.20	131.24	68793.39	1884.8
Apples and products^*∗*^	^n^	101.00	1.03	103.53	2.8
Pineapples and products^*∗*^	Pineapple, pulp, raw	104.65	0.99	103.61	2.8
Fruits, other	^o^	151.06	12.89	1946.39	53.3
Coffee and products^*∗*^	Coffee, instant, powder	3640.00	0.06	200.20	5.5
Cocoa beans and products^*∗*^	Beverages, cocoa mix, powder	712.00	3.68	2616.60	71.7
Tea (including mate)^*∗*^	Tea, infusion	18.00	0.05	0.90	0.00
Spices, other^*∗*^	Spices, mix, ground	1040.00	0.05	52.00	1.4
Beer	^p^	31.33	4.31	135.05	3.7
Beverages, fermented	Ovaltine beverage with skimmed milk (without sugar, fortified)	204.00	14.05	2866.20	78.5
Pig meat	Pork, meat, approximately 40% fat, boiled^*∗*^ (without salt)	230.77	1.04	240.00	6.6
Bovine meat	Beef, meat, 15–20% fat, boiled^*∗*^ (without salt)	254.00	1.23	312.42	8.6
Mutton and goat meat	Goat, meat, boiled^*∗*^ (without salt)	316.04	1.75	553.08	15.2
Poultry meat	Chicken, light meat, flesh and skin, boiled^*∗*^ (without salt)	128.00	7.09	906.88	24.8
Meat, other	Game meat, dried	923.00	4.86	4481.17	122.8
Cream^*∗*^	^q^	114.50	0.02	2.29	0.1
Butter, ghee	Cheddar	82.30	0.10	7.82	0.2
Fats, animals, raw	Margarine, fortified	18.00	0.20	3.60	0.1
Eggs	^r^	133.64	1.17	156.36	4.3
Milk excluding butter	Milk, cow, canned, evaporated	303.00	8.67	2627.01	72
Fishes	^s^	258.05	6.28	1619.89	44.4
Crustaceans^*∗*^	^t^	204.00	0.08	16.32	0.4
Cephalopods^*∗*^	^u^	454.50	0.10	43.18	1.2
**Total**					**9,086**

^a^Cassava, tuber, boiled (without salt), and cassava, tuber, dried. ^b^Cocoyam, tuber, boiled (without salt), and taro, tuber, boiled (without salt). ^c^Beans, liquid from stewed kidney beans; beans, baked, home prepared; peas, edible-podded, boiled, drained, without salt; peas, edible-podded, frozen, cooked, boiled, drained, with salt; peas, green, raw; and cowpea, boiled (without salt). ^d^Soyabean, boiled (without salt), and soyabean, combined varieties, boiled (Ghana) (without salt). ^e^Groundnut, shelled, dried, raw, and groundnut paste. ^f^Coconut, mature kernel, fresh, raw; coconut, immature kernel, fresh, raw; coconut, kernel, dried, raw; and coconut water. ^g^Tomato, red, ripe, boiled (without salt), and tomato paste, concentrated. ^h^Onion, raw, and onion, boiled (without salt). ^i^Cocoyam, leaves, boiled^*∗*^ (without salt); amaranth leaves, boiled (without salt); cabbage, raw; carrot, raw; eggplant, boiled (without salt); garlic, raw; lettuce, raw; okra fruit, boiled (without salt); peppers, chilli, raw; pepper, sweet, red, raw; pepper, sweet, red, boiled (without salt); pepper, sweet, green, raw; and pepper, sweet, green, boiled (without salt). ^j^Lemon, raw, and juice, lemon, unsweetened. ^k^Juice, grapefruit, canned, unsweetened, and grapefruit, pulp, raw. ^l^Banana, white flesh, raw, and banana, yellow flesh, raw. ^m^Plantains, cooked; plantains, green, fried; snacks, plantain chips, salted; plantain, ripe, boiled (without salt); and plantains, ripe, fried. ^n^Sweet apple, fruit, raw; juice, apple, canned or bottled; apple, with skin, raw; and apple, without skin, raw. ^o^Avocado, pulp, raw; mango, deep orange flesh; mango, orange flesh, raw; papaya, fruit, ripe, raw; and watermelon, fruit, raw. ^p^Beer European (4.4% alcohol); beer, millet (est. 3% alcohol); and beer, sorghum (est. 3% alcohol). ^q^Cream, whipping, 38% fat, and cream, 13% fat. ^r^Egg, chicken, boiled (without salt), and egg, chicken, fried. ^s^Anchovy, fillet, steamed (without salt); mackerel, grilled (without salt and fat); mudfish, grilled (without salt and fat); sardine, steamed (without salt); tilapia, grilled (without salt and fat); and tuna, grilled (without salt and fat). ^t^Spiny lobster, mixed species, cooked, moist heat, and crab, queen, cooked, moist heat. ^u^Octopus, common, cooked, moist heat, and squid, mixed species, cooked, fried. ^*∗*^K composition was sourced from the USDA National Nutrient Database for Standard Reference Software v.2.6.1, and the rest were from the FAO West African Food Composition Table. The K composition (mg per 100 g edible portion) was converted to mg per kg food intake by multiplying by 10.

**Table 2:** Extent of adequacy of individual K intake for different sex and age categories in Ghana based on 2010-2011 average food supply.

	Female		Male	
	19–50 years old	51+ years old	19–50 years old	51+ years old
SD_*D*10_	295.87	294.94	298.60	296.45
SD_*D*15_	441.01	440.38	442.84	441.40
*D*/SD_*D*10_	19.00	19.06	18.83	18.97
*D*/SD_*D*15_	12.75	12.77	12.70	12.74

*Note.* SD_*D*10_ denotes the daily variation in individual intake of K estimated at 10% standard deviation, while SD_*D*15_ denotes the estimate at 15%. Values used in the calculations: required intake = 2925 mg per day; standard deviations of the required intake at 10 and 15% (SD_r10_ and SD_r15_, respectively) = 292.5 mg per day and 438.75 mg per day, respectively.

## Data Availability

The food supply data used in the current study were retrieved from the Food Balance Sheet for Ghana, available at or accessible from the FAOSTAT website (http://www.fao.org/faostat/en/#home) or available upon request to the corresponding author. The K contents of the food components were obtained from either the West African Food Composition Table [26] or the United States Department of Agriculture-Agricultural Research Service (USDA-ARS) Nutrient Database for Standard Reference [27] (http://www.ars.usda.gov/nutrientdata; accessed on 15 April 2016).
